# Impact of glycosylated hemoglobin on early neurological deterioration in acute mild ischemic stroke patients treated with intravenous thrombolysis

**DOI:** 10.3389/fnagi.2022.1073267

**Published:** 2023-01-12

**Authors:** Lin Han, Zhangyan Hou, Mingwei Ma, Dongxue Ding, Dapeng Wang, Qi Fang

**Affiliations:** Department of Neurology, The First Affiliated Hospital of Soochow University, Suzhou, China

**Keywords:** acute mild ischemic stroke, intravenous thrombolysis, glycosylated hemoglobin, early neurological deterioration, functional outcomes

## Abstract

**Objective:**

In patients with acute mild ischemic stroke treated with intravenous thrombolysis, the relationship between chronic hyperglycemic status and their early neurological deterioration (END) and clinical outcomes is unclear. We attempted to analyze the relationship between glycated hemoglobin (HbA1c) levels and END and 90-day functional outcomes.

**Participants and methods:**

The research comprised 267 patients with acute mild ischemic stroke. The incidence of END and functional outcomes at 90 days were evaluated between subgroups. END was defined in this study as a rise of at least 1 point in the National Institutes of Health Stroke Scale (NIHSS) score within 72 h of admission, with an excellent outcome of a modified Rankin Scale (mRS) score of 0–1 at 90 days following stroke beginning. The association between HbA1c and END, and clinical outcomes in patients with mild stroke, was assessed by logistic regression after adjusting for confounding factors. In addition, we used receiver operating characteristic (ROC) curves to predict the predictive value of HbA1c for the incidence of END.

**Results:**

There were 38 patients who suffered END and 105 patients who had disabled functional outcomes at 90 days. In multivariate analysis, elevated HbA1c levels were associated with END (adjusted OR = 1.476; 95% CI: 1.129–1.928; *p* = 0.004). With HbA1c greater than 7.75%, the ROC curve predicted a higher risk of END. However, they were not associated with patients’ functional outcomes at 90 days.

**Conclusion:**

HbA1c levels were an independent predictor of END in patients with mild stroke, while there was no effect on functional outcomes at 90 days. The impact of HbA1c on functional prognosis may be a contributing factor rather than a direct factor.

## Introduction

Mild ischemic stroke accounts for more than a half of all ischemic stroke patients (Reeves et al., 2013). However, patients who initially present with mild symptoms may eventually develop severe disability, and recent evidence suggests that early neurological deterioration (END) is one of the major predictors of poor prognosis (Seners et al., 2014; Mazya et al., 2018; Saleem et al., 2020). Although the effectiveness and safety of intravenous thrombolytic therapy in patients with mild neurological symptoms of ischemic stroke remains controversial, in clinical practice, 40–50.5% of patients receive intravenous thrombolytic therapy (Duan et al., 2020; Saber et al., 2020; Wang et al., 2021). Moreover, within a 4.5-h window of stroke onset, international guidelines recommend intravenous thrombolysis for disabling ischemic mild stroke (Powers et al., 2018; Berge et al., 2021). Recently, several multicenter studies of mild stroke have reported that END may occur in 10–30% of patients, and the majority are associated with recanalization occlusion (National Institute of Neurological Disorders and Stroke rt-PA Stroke Study Group, 1995; Boulenoir et al., 2021; Seners et al., 2021). Some studies have aimed to detect predictors of END in all patients with ischemic stroke, which may help in early identification of patients at a risk of progression after stroke onset. But few studies have focused on patients with mild stroke. Therefore, it is of great importance to identify factors associated with END in patients with acute mild stroke.

Previous research found that diabetes was an independent predictor of END in minor strokes (Ferrari et al., 2010). Poor glucose control is associated with poor functional outcomes after stroke, suggesting that long-term impaired glucose metabolism is associated with the functional prognosis of ischemic stroke patients (Lattanzi et al., 2016). Merlino et al. (2021) found that stress hyperglycemia was associated with poor outcomes after intravenous thrombolysis in 414 patients with acute ischemic stroke, regardless of diabetic status. High blood glucose is associated with brain–blood barrier breakdown, brain edema, and increased apoptosis (Song et al., 2003; Chiu et al., 2013). In addition, hyperglycemia accelerates damage to neurons in hypoxic brain tissue (Luitse et al., 2012). Nevertheless, recently, the effect of hyperglycemia on END in patients with acute ischemic stroke has been considered controversial, it may be a contributing factor rather than a direct cause (Seners et al., 2015). Although hyperglycemia on admission may reflect abnormal glucose metabolism already present before the onset of stroke, it may also be the result of acute stress and inflammatory response after acute ischemic stroke. Hence, to more accurately assess the impact of pre-stroke hyperglycemic status on acute ischemic stroke, prior to the stroke, blood glucose levels must be monitored for an extended length of time. Chronic hyperglycemia, also known as pre-stroke glycemic control, is usually assessed by glycated hemoglobin (HbA1c), which is a reliable indicator of average blood glucose levels over a 2–3 month period (American Diabetes, 2014).

The predictive value of HbA1c for the incidence of END and functional outcomes at 90 days in mild stroke patients remains unclear. In this study, we aimed to evaluate the impact of chronic hyperglycemic status on END and clinical outcomes at 90 days in acute mild ischemic stroke patients treated with intravenous recombinant tissue-type plasminogen activator (rt-PA).

## Materials and methods

### Study population

**Figure 1:**
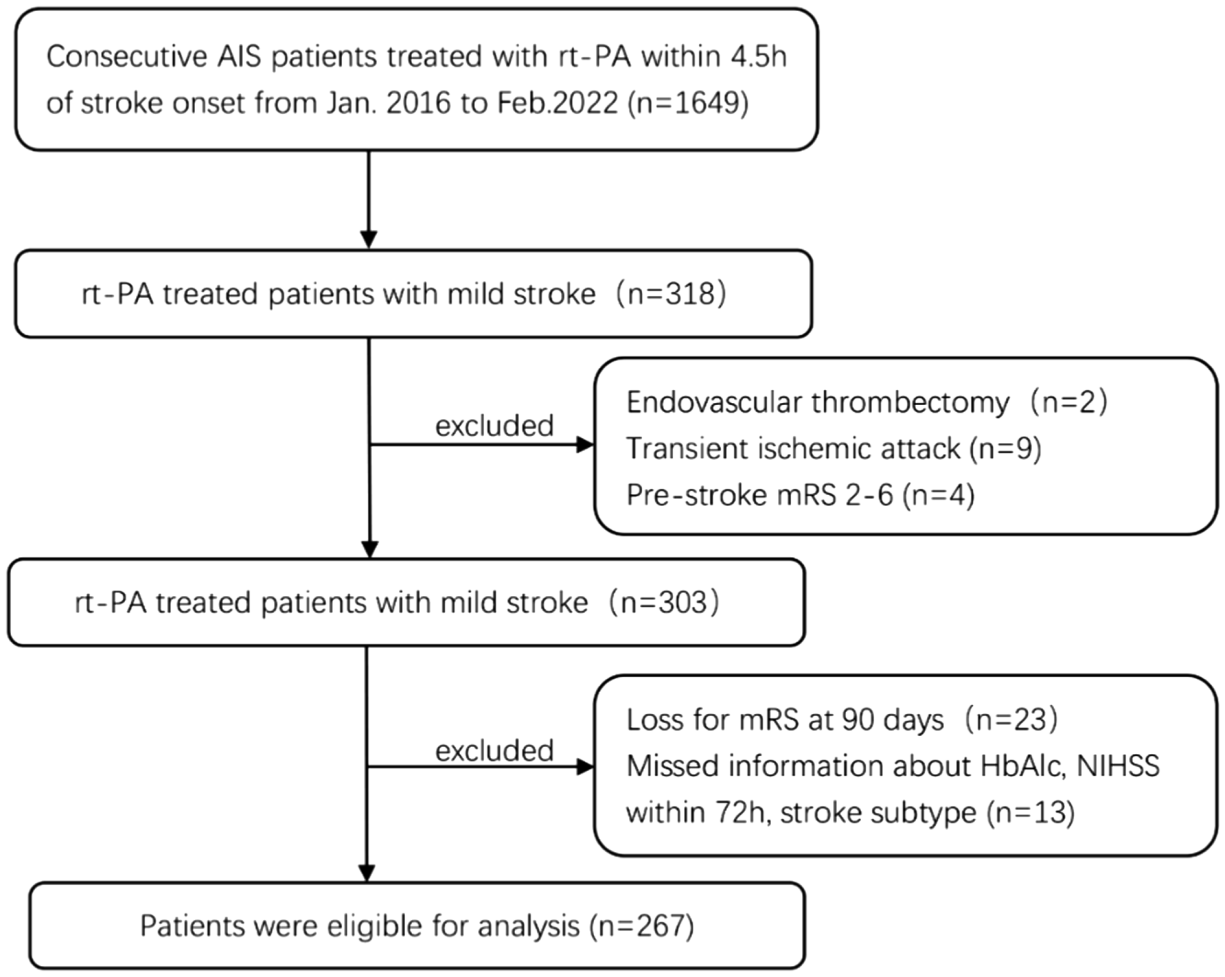
Study inclusion flow chart. AIS, acute ischemic stroke; rt-PA, recombinant tissue plasminogen activator; mRS, modified Rankin Scale Score; HbA1c, glycated hemoglobin; NIHSS, National Institute of Health Stroke Scale.

The present study performed a retrospective analysis of prospectively collected data from the Stroke Center of the First Hospital Affiliated of Soochow University between January 2016 and February 2022. All enrolled patients suffered from acute cerebral ischemic stroke within 4.5 h of symptom onset and were treated with intravenous rt-PA according to the clinical judgment of trained neurologists. The inclusion criteria were as follows: (1) baseline admission National Institutes of Health Stroke Scale (NIHSS) score ≤ 5; (2) patients with a modified Rankin Scale (mRS) score ≤1 prior to stroke onset; (3) no further endovascular treatment. The exclusion criteria were as follows: (1) diagnosis of malignant brain tumor, severe hepatic or renal dysfunction, or other severe systemic diseases; (2) incomplete clinical data. Among the 318 patients with mild stroke, two patients who received endovascular therapy after intravenous thrombolysis, nine patients were transient ischemic attacks, and four patients with pre-onset mRS score >1, were excluded. While 23 patients were lost to follow-up or refused to offer information after discharge, we further excluded 13 patients without information about HbA1c, NIHSS within 72 h, or stroke subtype, for a final sample of 267 patients with mild stroke (Figure 1).

This study was approved by the Ethics Committee of the First Hospital Affiliated to Soochow University (NO. 2022403). Written informed consent for participation was not required for this study in accordance with the national legislation and the institutional requirements.

### Clinical data

The following data were collected for all patients: demographics, past medical history, and vascular risk factors, including a medical history of hypertension, diabetes, atrial fibrillation, coronary artery disease, and current smoking. Baseline stroke severity was assessed with the NIHSS (Brott et al., 1989). Fasting blood samples are routinely collected in the morning after intravenous thrombolysis; the stroke subtype was classified according to the Trial of Org 10172 in Acute Stroke Treatment (TOAST) criteria (Adams et al., 1993). Diabetes was defined according to patients’ self-reported history or the use of hypoglycemic drugs or insulin. END was defined as an NIHSS score increased ≥1 during the first 72 h compared with the initial NIHSS score. Functional outcomes were determined at 90 days after stroke onset. Excellent functional outcomes were defined as a 90-day mRS score of 0 to 1, while disabled functional outcomes were defined as an mRS score of ≥2. Intracranial hemorrhage (ICH) was confirmed by CT, asymptomatic intracranial haemorrhage (ASICH) and symptomatic intracranial haemorrhage (SICH) based on presence or absence of exacerbation of neurological deficits. According to ECASS II, it defines SICH as ICH with worsening in NIHSS greater than or equal to 4 points (Hacke et al., 1998). In addition, we did not exclude patients with END due to haemorrhagic transformation. Neurological deficits were assessed at admission and within 72 h after intravenous rt-PA by two board-certified neurologists who were unaware of the clinical data.

### Statistical analyses

The data was tested for normal distribution using the Kolmogorov–Smirnov test. Continuous variables were described as mean ± SD or median (interquartile range, IQR), the student’s *t*-test or Mann–Whitney *U* test was used to test the difference between groups, according to normality. Categorical variables were expressed as numbers and percentages and analyzed using the chi-square or Fisher’s exact tests, when appropriate. Multivariable binary logistical regression was used to analyze the predictive value of the HbAlc for END and unfavorable functional outcomes at 90 days for all patients. Logistic regression models were adjusted for potential confounders, including age, sex, NIHSS on admission, fibrinogen, hs-CRP, fasting glucose, diabetes, and stroke subtype. Collinear diagnosis was performed for diabetes, fasting glucose and HbA1c, with VIF less than 10 indicating no collinearity. A receiver operating characteristics (ROC) curve was generated to examine the predictive value of HbA1c for END, based on the area under the ROC curve (AUC). The two-tailed *p*-values of <0.05 were considered statistically significant. All statistical analyses were performed using SPSS (IBM SPSS Statistics for Windows, version 26.0; SPSS Inc., Chicago, IL, United States), and figures were prepared using GraphPad Prism 9.

## Results

### Baseline characteristics

A total of 267 patients with mild stroke were included in this study, with a median age of 66 years, and 182 (68.2%) of them were men. The median NIHSS score on admission was 3. END occurred in 38 (14.2%) patients, which is similar to the previous study (Seners et al., 2021) and 105 (39.3%) experienced disabled functional outcomes. Table 1 summarizes the baseline clinical and biochemical characteristics, grouped by the occurrence and non-occurrence of END. Compared to the non-END group, we found that patients in the END group had higher NIHSS scores at 7 days after stroke onset, poorer 90-day functional outcomes. This may be related to higher levels of high-sensitivity C-reactive protein, fibrinogen, and glucose in the END group.

**Table 1:** Baseline clinical and biochemical characteristics, according to END.

	Total (*n* = 267)	END (*n* = 38)	Non-END (*n* = 229)	*p*-Value
*Characteristics*				
Age, year, median (IQR)	66.0 (57.0–75.0)	67.0 (57.5–74.0)	66.0 (57.0–75.0)	0.860
Male sex, *n* (%)	182 (68.2)	24 (63.2)	158 (69.0)	0.474
Baseline SBP, mean ± SD, mmHg	157.36 ± 26.01	162.21 ± 29.79	156.55 ± 25.32	0.215
Baseline DBP, median (IQR), mmHg	89.00 (78.00–97.00)	89.00 (79.75–96.00)	89.00 (78.00–97.50)	0.525
Admission NIHSS score, median (IQR)	3 (2–4)	3 (2–4)	3 (2–4)	0.854
NIHSS on 7 days, median (IQR)	1 (0–2)	4.5 (2–9)	1 (0–1)	0.000
mRS at 90 days, median (IQR)	1 (1–2)	3 (2–4)	1 (0.5–2)	0.000
*Clinical status before admission*				
Hypertension, *n* (%)	198 (74.2)	27 (71.1)	171 (74.7)	0.637
Diabetes mellitus, *n* (%)	73 (27.3)	20 (52.6)	53 (23.1)	0.001
Hyperlipidemia, *n* (%)	22 (8.2)	3 (7.9)	19 (8.3)	0.616
Atrial fibrillation, *n* (%)	47 (17.6)	6 (15.8)	41 (17.9)	0.751
Coronary heart disease, *n* (%)	25 (9.4)	1 (2.6)	24 (10.5)	0.099
Current smoking, *n* (%)	112 (41.9)	19 (50.0)	93 (40.6)	0.277
*TOAST classification*				0.311
Large artery atherosclerosis, *n* (%)	136 (50.9)	24 (63.2)	112 (48.9)	
Cardiogenic embolism, *n* (%)	37 (13.9)	3 (7.9)	34 (14.8)	
Small vessel occlusion, *n* (%)	70 (26.2)	7 (18.4)	63 (27.5)	
Other/Undetermined, *n* (%)	24 (9.0)	4 (10.5)	20 (8.7)	
*Biochemical variables*				
hs-CRP, median (IQR), mg/L	3.76 (1.47–8.14)	7.00 (1.60–12.49)	3.49 (1.44–6.73)	0.019
Fbg, median (IQR), g/L	2.67 (2.25–3.20)	3.14 (2.51–3.40)	2.63 (2.23–3.14)	0.005
Fasting Glu, median (IQR), mmol/L	5.34 (4.71–6.99)	6.34 (5.45–8.52)	5.19 (4.67–6.62)	0.000
LDL-c, median (IQR), mmol/L	2.78 (2.19–3.42)	2.77 (2.24–3.81)	2.78 (2.18–3.40)	0.362
TC, median (IQR), mmol/L	4.45 (3.82–5.18)	4.49 (4.06–5.75)	4.44 (3.79–5.12)	0.082
TG, median (IQR), mmol/L	1.38 (1.00–1.95)	1.43 (1.00–2.11)	1.37 (0.99–1.95)	0.720
Cr, median (IQR), μmol/L	67.70 (58.30–78.00)	66.25 (48.05–76.93)	68.00 (59.50–78.00)	0.269
HbAlc, median (IQR), %	6.20 (5.80–6.90)	7.05 (6.07–10.32)	6.20 (5.80–6.74)	0.000
hcy, median (IQR), μmol/L	10.30 (8.50–13.32)	9.50 (7.83–13.93)	10.50 (8.70–13.31)	0.398
*Intracerebral hemorrhage*				0.123
ASICH, *n* (%)	6 (2.2)	1 (2.6)	5 (2.2)	
SICH, *n* (%)	9 (3.4)	3 (7.9)	6 (2.6)	

IQR, interquartile range; SD, standard deviation; SBP, systolic blood pressure; DBP, diastolic blood pressure; NIHSS, National Institute of Health Stroke Scale; mRS, modified Rankin Scale; hs-CRP, hypersensitive C-reactive protein; Fbg, fibrinogen; Glu, glucose; LDL-C, low-density lipoprotein cholesterol; TC, total cholesterol; TG, triglyceride; Cr, creatinine; HbA1c, glycated hemoglobin; hcy, homocysteine; ASICH, asymptomatic intracerebral hemorrhage; SICH, symptomatic intracerebral hemorrhage.

**Figure 2:**
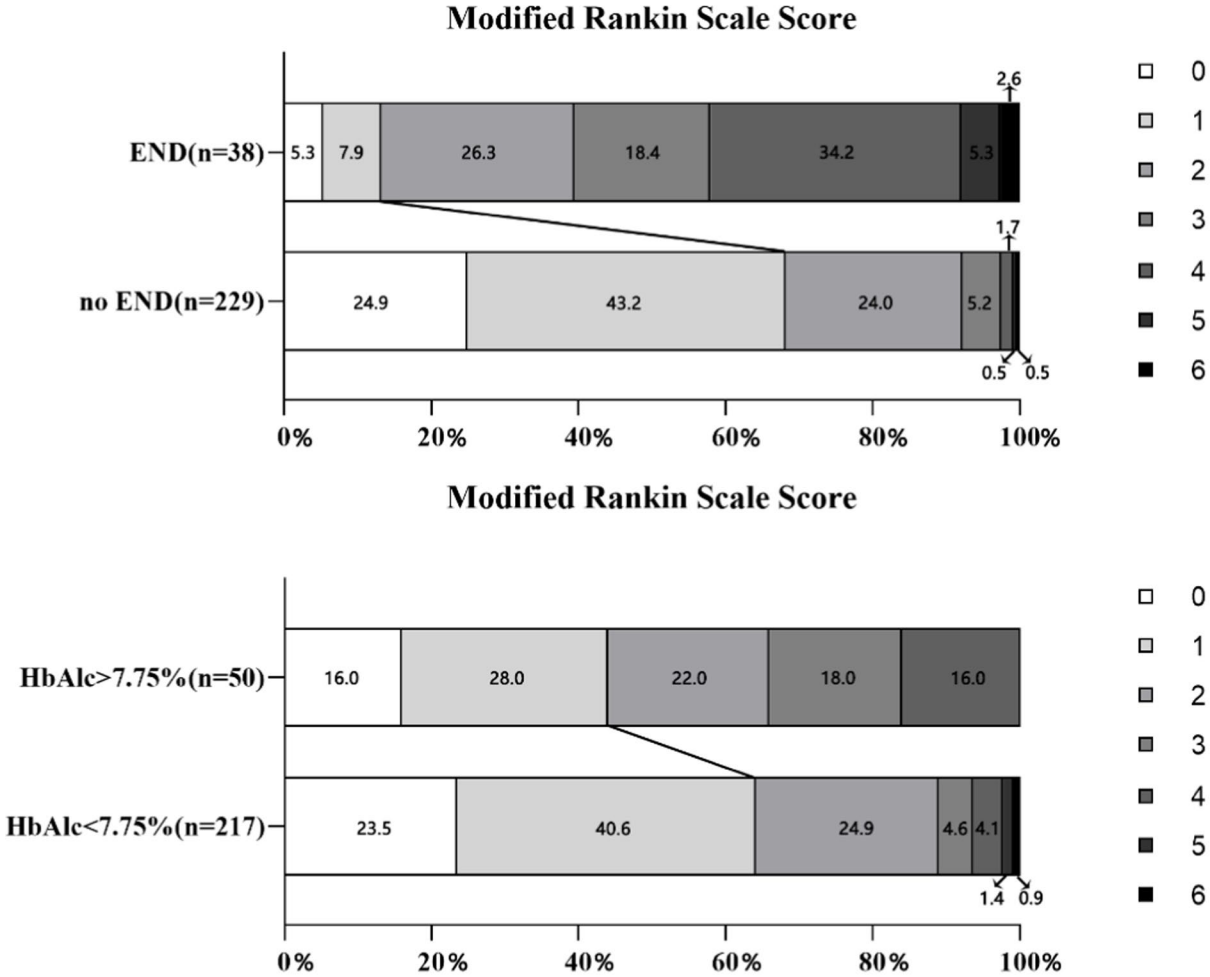
Distribution of scores on the modified Rankin Scale at 90 days.

We also stratified the enrolled patients by their functional outcomes. Patients with excellent outcomes tended to have lower baseline NIHSS scores, no diabetes, or weaker inflammatory responses, and lower fibrinogen levels. Notably, HbA1c did not affect functional outcomes, whereas early neurological deterioration severely affected patients’ functional recovery (Figure 2). The baseline and clinical features of the patients stratified according to the primary outcomes are summarized in Table 2. In addition, another notable result was the lower risk of ICH in our patients with mild stroke, the incidence of SICH was 3.4%. However, there is no statistically significant difference in intracranial hemorrhage between the END and non-END groups, the excellent and disabled functional outcomes groups.

**Table 2:** Baseline clinical and biochemical characteristics, stratified for functional outcomes.

	Excellent outcomes (*n* = 162)	Disabled outcomes (*n* = 105)	*p*-Value
	mRS ≤ 1 (90 days)	mRS > 1 (90 days)	
*Characteristics*			
Age, y, median (IQR)	65.0 (56.0–73.3)	69.0 (58.0–75.5)	0.069
Male sex, *n* (%)	108 (66.7)	74 (70.5)	0.514
Baseline SBP, mean ± SD, mmHg	156.06 ± 27.33	159.37 ± 23.82	0.31
Baseline DBP, median (IQR), mmHg	88.00 (77.75–96.00)	89.00 (80.00–99.00)	0.271
Admission NIHSS score, median (IQR)	3.0 (2.0–3.3)	3 (2–4)	0.011
*Clinical status before admission*			
Hypertension, *n* (%)	117 (72.2)	81 (77.1)	0.37
Diabetes mellitus, *n* (%)	36 (22.2)	37 (35.2)	0.02
Hyperlipidemia, *n* (%)	15 (9.3)	7 (6.7)	0.452
Atrial fibrillation, *n* (%)	29 (17.9)	18 (17.1)	0.874
Coronary heart disease, *n* (%)	16 (9.9)	9 (8.6)	0.721
Current smoking, *n* (%)	63 (38.9)	49 (46.7)	0.208
*TOAST classification*			0.01
Large artery atherosclerosis, *n* (%)	71 (43.8)	65 (61.9)	
Cardiogenic embolism, *n* (%)	23 (14.2)	14 (13.3)	
Small vessel occlusion, *n* (%)	52 (32.1)	18 (17.1)	
Other/Undetermined, *n* (%)	16 (9.9)	8 (7.8)	
END, *n* (%)	5 (3.1)	33 (31.4)	0.000
*Biochemical variables*			
hs-CRP, median (IQR), mg/L	3.35 (1.44–6.39)	5.78 (1.57–11.86)	0.012
Fbg, median (IQR), g/L	2.62 (2.17–3.08)	2.84 (2.37–3.35)	0.047
Fasting Glu, median (IQR), mmol/L	5.20 (4.70–6.57)	5.58 (4.78–7.90)	0.074
LDL-c, median (IQR), mmol/L	2.80 (2.19–3.40)	2.69 (2.19–3.45)	0.953
TC, median (IQR), mmol/L	4.46 (3.83–5.17)	4.39 (3.79–5.24)	0.907
TG, median (IQR), mmol/L	1.41 (0.96–2.03)	1.36 (1.04–1.88)	0.9
Cr, median (IQR), μmol/L	66.95 (58.23–77.85)	69.40 (58.60–79.00)	0.616
HbAlc, median (IQR), %	6.20 (5.80–6.74)	6.30 (5.80–7.85)	0.129
hcy, median (IQR), μmol/L	10.00 (8.48–13.30)	10.70 (8.65–13.46)	0.289
*Intracerebral hemorrhage*			0.155
ASICH, *n* (%)	5 (3.1)	1 (1.0)	
SICH, *n* (%)	3 (1.9)	6 (5.7)	

IQR, interquartile range; SD, standard deviation; SBP, systolic blood pressure; DBP, diastolic blood pressure; NIHSS, National Institute of Health Stroke Scale; mRS, modified Rankin Scale; END, early neurological deterioration; hs-CRP, hypersensitive C-reactive protein; Fbg, fibrinogen; Glu, glucose; LDL-C, low-density lipoprotein cholesterol; TC, total cholesterol; TG, triglyceride; Cr, creatinine; HbA1c, glycated hemoglobin; hcy, homocysteine; ASICH, asymptomatic intracerebral hemorrhage; SICH, symptomatic intracerebral hemorrhage.

### Association of the HbA1c with END and functional outcomes in multivariate analysis

We also examined the relationship between HbA1c and END and functional outcomes at 90 days using HbA1c as a continuous variable. As demonstrated in Table 3, greater HbA1c levels were linked with END in general, with an adjusted advantage ratio (OR) of 1.476 (95% CI: 1.129–1.928). However, after multifactorial adjustment, high or low HbA1c had no effect on the functional outcomes at 90 days. We found that admission NIHSS and END were all independent factors influencing the 90-day functional outcomes.

**Table 3:** Logistic regression analysis showing the impact of HbAlc on END and functional outcomes in acute mild ischemic stroke patients treated with intravenous thrombolysis.

	Crude results		Adjusted results	
	OR (95% CI)	*p*-Value	OR (95% CI)	*p*-Value
END	1.476 (1.245,1.750)	0	1.476 (1.129,1.928)	0.004
Functional outcomes	1.200 (1.037,1.389)	0.014	0.975 (0.747,1.271)	0.85

END as the dependent variable, adjusted for age, sex, NIHSS on admission, Fbg, hs-CRP, fasting glucose, diabetes and stroke subtype. Functional outcome as the dependent variable, adjusted for age, sex, NIHSS on admission, Fbg, hs-CRP, fasting glucose, diabetes, stroke subtype, and END. END, early neurological deterioration; OR, odds ratio; CI, confidence interval, NIHSS, National Institute of Health Stroke Scale; Fbg, fibrinogen; hs-CRP, hypersensitive C-reactive protein.

### Optimal predictive value of HbA1c for END

Receiver operating characteristic (ROC) curve analysis was used to evaluate the predictive values of HbA1c for END. Increased HbA1c was associated with END with an area under the curve of 0.676 (95% CI, 0.572–0.779; *p* = 0.001). The optimal cutoff value of HbA1c to distinguish END was 7.75, with 47.4% sensitivity and 86.0% specificity. In addition, we found that favorable outcomes occurred more often in the HbA1c ≤ 7.75% group than in the HbA1c > 7.75% group. Nevertheless, there were no patients with severe disability or even death in the HbA1c > 7.75% group at 90 days (Figure 2).

## Discussion

In the present study, we showed that high HbA1c levels were independently associated with END in patients with mild stroke after intravenous thrombolysis. ROC analysis revealed that the optimal predictive value of HbA1c was 7.75% for END. These results suggest that END is more likely to occur after stroke if blood glucose levels are poorly controlled prior to the stroke. In other words, greater HbA1c levels increase the odds of the develop the END in mild stroke patients. To the best of our knowledge, this is the first study examining the relationship between HbA1c and END in patients with acute mild ischemic stroke. This may help identify high-risk groups for END in specific patient populations.

Mechanistically, the hyperglycemic condition before reperfusion may function as a fibrinolytic inhibitor, reducing the efficiency of rt-PA (Alvarez-Sabin et al., 2003). Second, prolonged hyperglycemia may impair neurological recovery following ischemia damage in individuals receiving intravenous rt-PA (Masrur et al., 2015). Endothelial dysfunction, free radical generation, and impairment of the brain’s autoregulatory system may all contribute to reperfusion damage after successful recanalization in patients receiving intravenous thrombolysis (Martini and Kent, 2007; Suh et al., 2008; Choi et al., 2018; Diprose et al., 2020). It is also likely that persistent hyperglycemia causes microvascular remodeling, which impairs collateral circulation (Hou et al., 2013). Moreover, the chronic hyperglycemic state may be a condition in which the vessel walls become weak and alter the blood–brain barrier permeability, thereby increasing the risk of brain edema or reperfusion injury (Dua et al., 2010; Desilles et al., 2017; Shukla et al., 2017).

As for HbA1c levels did not affect functional prognosis, a possible explanation is that poor glycemic status may be a marker of stroke severity rather than a predictor of subsequent functional recovery, that is, a high HbA1c level may lead to END rather than directly affect functional outcomes. It may be related to the tolerance mechanism in patients with chronic hyperglycemia, which offsets adverse metabolic effects. Similar findings were observed in the stress hyperglycemia research, which found that stroke patients with stress hyperglycemia had a greater likelihood of experiencing an END but not a higher risk of poor outcome (Wang et al., 2022). As the END is strongly associated with poor functional prognosis (Seners et al., 2014; Mazya et al., 2018; Saleem et al., 2020), rigid blood glucose management before a stroke may have a beneficial effect on clinical outcomes. In addition, an interesting finding in our study was the relatively large number of diabetic patients in the unfavorable functional outcomes group. It is similar to previous studies that reported poorer neurological recovery and more severe disability in diabetic patients compared to non-diabetic patients (Megherbi et al., 2003; Roquer et al., 2014). Accordingly, current guidelines recommend a target HbA1c <7.0% to reduce vascular complications of diabetes (Kleindorfer et al., 2021). Despite this, it has been shown that increased HbA1c levels are an independent predictor of unfavorable clinical outcomes in patients with ischemic stroke (Lei et al., 2015), which might be owing to the distinct research group, as our study only included patients with mild stroke.

It is a worthwhile question to think about how glucose management should be performed in patients with mild stroke in the acute phase. In a large health system-based cohort study, lower HbA1c and higher HbA1c levels were found to be associated with a higher risk of stroke, forming a U-shaped curve. Intensive glucose reduction and poor glycemic control may be associated with an increased risk of stroke in patients with diabetes (Shen et al., 2020). Another multicenter randomized clinical trial showed that intensive glycemic control in acute ischemic stroke patients with hyperglycemia did not improve their functional outcomes significantly. This may be attributed to the higher incidence of hypoglycemia in the intensive treatment group (Johnston et al., 2019). As a result, in order to minimize the occurrence of END events, it is crucial to focus on stroke patients with high HbA1c and to administer modest glucose-lowering medication in the acute period. In contrast, it is critical to check for glucose metabolic status before a stroke starts in order to give rigorous glycemic control by lifestyle modifications and medication, which may prevent stroke occurrences and react better to reperfusion treatment in the acute phase of stroke.

Consistent with previous reports, the probability of haemorrhagic transformation after intravenous thrombolysis is low in mild stroke patients, this may suggest that END was more ischemic in origin (Mazya et al., 2018; Seners et al., 2021). Additionally, the low incidence of SICH indicates that intravenous rt-PA therapy may be safe and effective in patients with mild stroke (Xiong et al., 2021). Therefore, the difference between the higher rate of END and the lower risk of intracranial hemorrhage in mild stroke patients receiving intravenous thrombolysis, especially those with high HbA1c levels, requires that we treat these patients more actively. For example, lowering blood sugar properly (Wang et al., 2022) and early anti-platelet therapy (Li et al., 2022).

This study has several limitations. First, our statistical power is relatively limited due to the rather small number of patients who developed END in mild stroke patients treated with intravenous thrombolysis. Second, this is a single-center, retrospective study, and this study may be subject to recruitment bias. Third, we did not further compare with mild stroke patients who did not receive intravenous thrombolytic therapy. Because of these limitations, the results of our study should be interpreted with caution. Further prospective and randomized studies are needed to confirm our results.

## Conclusion

In conclusion, our study suggests that higher HbA1c levels are an independent predictor of END in mild stroke patients treated with intravenous thrombolysis, whereas there is no effect on functional outcome at 90 days. According to our findings, the risk of END is particularly significant when HbA1c exceeds 7.75%, which requires aggressive glucose-lowering therapy, and it seems reasonable to maintain HbA1c levels below 7%.

## Data availability statement

The original contributions presented in the study are included in the article/Supplementary material, further inquiries can be directed to the corresponding authors.

## Ethics statement

The studies involving human participants were reviewed and approved by the Ethics Committee of the First Hospital Affiliated to Soochow University (No. 2022403). Written informed consent for participation was not required for this study in accordance with the national legislation and the institutional requirements.

## Author contributions

QF and DW conceived and designed the research. DD analyzed the data. LH drafted the manuscript. LH, ZH, MM, and DW collected the data and performed the research. All authors reviewed and edited the manuscript and approved the final version of the manuscript.

## Funding

This study was supported by the National Natural Science Foundation of China (Nos. 82071300 to QF and 82001219 to DD) and the Natural Science Foundation of Jiangsu Province (BK20190183).

## Conflict of interest

The authors declare that the research was conducted in the absence of any commercial or financial relationships that could be construed as a potential conflict of interest.

## Publisher’s note

All claims expressed in this article are solely those of the authors and do not necessarily represent those of their affiliated organizations, or those of the publisher, the editors and the reviewers. Any product that may be evaluated in this article, or claim that may be made by its manufacturer, is not guaranteed or endorsed by the publisher.

## Supplementary material

The Supplementary material for this article can be found online at: https://www.frontiersin.org/articles/10.3389/fnagi.2022.1073267/full#supplementary-material
