# Supplementary figures and images for: Impact of glycosylated hemoglobin on early neurological deterioration in acute mild ischemic stroke patients treated with intravenous thrombolysis
Source: Front Aging Neurosci. 2023 Jan 12;14:1073267. doi: 10.3389/fnagi.2022.1073267 (PMC9877605; doi:10.3389/fnagi.2022.1073267)

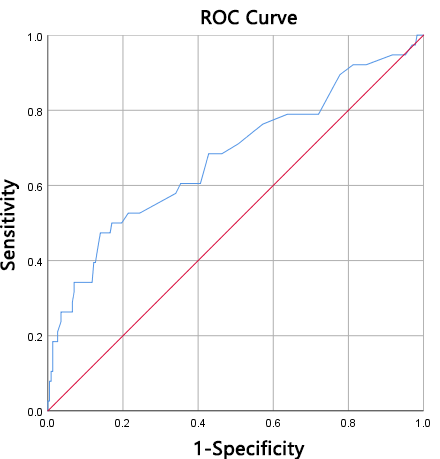

Supplement: Supplementary file 1 [file Image_1.tif]
